# Public Attitudes Toward Policy Instruments for Flood Risk Management

**DOI:** 10.1007/s00267-023-01848-3

**Published:** 2023-07-01

**Authors:** Jonathan Raikes, Daniel Henstra, Jason Thistlethwaite

**Affiliations:** 1 Sustainability Research Centre, University of the Sunshine Coast, 90 Sippy Downs Dr., Sippy Downs, QLD 4556 Australia; 2 School of Environment, Enterprise and Development, University of Waterloo, 200 University Ave W, Waterloo, ON N2L 3G1 Canada; 3 Department of Political Science, University of Waterloo, 200 University Ave W, Waterloo, ON N2L 3G1 Canada

**Keywords:** Public opinion, Flood risk management, Social acceptability, Policy instruments

## Abstract

Effective flood risk management (FRM) requires a mix of policy instruments that reduces, shares, and manages flood risk. The social acceptability of these policy instruments—the degree of public support or opposition to their use—is an important consideration when designing an optimal mix to achieve FRM objectives. This paper examines public attitudes toward FRM policy instruments based on a national survey of Canadians living in high-risk areas. Respondents were asked their views on flood maps, disaster assistance, flood insurance, flood risk disclosure and liability, and property buyouts. The results indicate that all five policy instruments have high social acceptability, but they must be calibrated to ensure access to flood risk information and achieve a fair distribution of FRM costs among key stakeholders.

## Introduction

Flooding is one of the most significant global hazards and its impacts are worsening due to climate change, the location and expansion of urban settlements in high-risk areas, and increasing socioeconomic vulnerability (Hirabayashi et al. 2013; Kundzewicz et al. 2019; Yamazaki et al. 2018). Flood risk management (FRM) is a strategic approach that involves sharing responsibility with those who contribute to flood risk, increasing the participation of diverse stakeholders in decision-making, and allocating resources across a broad portfolio of measures to reduce, share, and manage flood risk (Hegger et al. 2018; Martinez et al. 2021).

Governments have access to a broad range of *policy instruments*—tools and mechanisms to achieve policy objectives—that they can employ to achieve FRM objectives (Verweij et al. 2021; Glaus 2021). Historically, governments favored structural controls, such as levees, dams, and dikes, which aimed to physically control the flow of water (Kundzewicz et al. 2018). In many countries, the central focus of FRM has now shifted toward non-structural instruments, such as flood maps, risk assessments, regulations, flood insurance, and disaster assistance, which are intended to minimize the exposure of structures and assets and to reduce vulnerability of people by influencing social behavior (Kelman 2013; Boyd and Markandya 2021).

The selection of FRM instruments is often criticized for emphasizing technical analysis without acknowledging the social importance of public support for implementing policy (de Moel et al. 2009; Dieperink et al. 2016; Driessen et al. 2016; Kundzewicz et al. 2018). *Social acceptability*—the degree of public support for, or opposition to, the policy instruments selected to achieve FRM objectives—has received scant attention in scholarly research, despite the fact that it can have a significant influence on the effectiveness of FRM (Buchecker et al. 2016; Cass et al. 2022). High social acceptability, as reflected through positive public opinions and attitudes, gives politicians greater confidence to implement and sustain FRM policies, while providing the legitimacy to enforce compliance with more coercive instruments (de Groot and Schuitema 2012; Capano and Lippi 2017). By contrast, low social acceptability reduces the effectiveness of policy instruments, while also posing significant reputational risks to policymakers (Burton and Mustelin 2013; Leiss and Larkin 2019; Nilsson et al. 2016). Social acceptability is important because FRM seeks to diversify responsibility away from governments to other stakeholders, including citizens, urban planners, emergency management officials, insurers, and realtors (Raikes et al. 2019). Moreover, non-structural instruments need a high level of social acceptability because their effectiveness depends on behavioral change among citizens and stakeholders.

This paper analyzes the social acceptability of different FRM policy instruments based on a national survey of Canadians living in high-risk flood areas. Specifically, it examines public attitudes on flood mapping, disaster assistance arrangements, disclosure and liability, homeowner flood insurance, and property buyouts. The findings contribute to a broader understanding of the potential efficacy of non-structural policy instruments for managing flood risk based on their degree of social acceptability. Key considerations for instrument design and decision-making are discussed.

The paper begins by discussing social acceptability as it is conceived in public policy literature. It then contextualizes FRM in Canada and harnesses existing literature to describe and explain the dynamics surrounding the five policy instruments. Third, the paper describes the research methods used to collect and analyze public attitudes toward the FRM policy instruments. Section *Methods* presents and synthesizes the national survey results. The paper concludes with a broader discussion on key considerations for FRM policy design and implementation based on these findings.

## Social Acceptability and Public Policy

Over the last decade, scholars and practitioners have devoted increasing attention to the social acceptability of public policy instruments, in recognition that the degree of public support for the tools employed influences whether a policy will be effective and legitimate in managing complex problems (Wicki et al. 2019; Cunningham et al. 2016; Verlynde et al. 2019; Bazart et al. 2020; Vona 2019; Mallette et al. 2021; Capano and Lippi 2017). Specifically, it has been found that the social acceptability of a policy intervention is influenced by the context of the policy problem, the receptiveness of key stakeholders to what the intervention is designed to achieve, and the distribution of roles and responsibilities associated with the policy instrument’s implementation (Dermont et al. 2017; Paul and Milman 2017).

In the context of FRM, it is argued that policy instruments are more likely to be socially acceptable (and therefore more effective) when responsibilities associated with the instrument are distributed among multiple stakeholders (e.g., households, governments, industry, civil society), the instrument’s financial impacts on property values are minimal, and controls on individual choices are perceived as low (Mallette et al. 2021; Persson et al. 2021; Drews and van den Bergh 2016).

## Study Context

Flooding is a significant risk facing many communities and households in Canada. According to the Canadian Disaster Database, there were 103 significant flood disasters between 2000 and 2019 (Public Safety Canada 2021). Flood risk is highly concentrated in “designated flood risk areas”—lands that are subject to recurrent and severe flooding—which can be found in every province (Natural Resources Canada and Public Safety Canada 2017). These areas are largely situated along rivers and coasts, but urban (pluvial) flooding is growing as a significant source of risk (Gaur et al. 2019; Sandink 2016).

Canada is a federation, in which responsibilities for FRM are divided between three levels of government (Golnaraghi et al. 2020). The federal Government of Canada generates weather and geospatial data to support flood mapping and decision-making, provides economic resources to mitigate and recover from flood risk, and develops broad policy frameworks that require collaboration with lower levels of government to implement. The regional governments—ten provinces and three territories—set regulatory standards for land use planning, enforce building and infrastructure standards, set expectations for municipal FRM, and provide disaster financial assistance for post-flood recovery. Municipal governments implement FRM by managing stormwater run-off, implementing flood defences, and planning for flood-related emergencies.

## FRM Policy Instruments

Faced with the escalating costs of replacing structural protections, and recognizing their growing economic liability associated with disaster financial assistance, both the federal and provincial governments have shown greater interest in non-structural instruments for FRM, particularly in designated flood risk areas (Golnaraghi et al. 2020). Henstra and Thistlethwaite (2017) identified seventeen non-structural policy instruments used by Canadian governments to reduce, share, and manage flood risk, and they noted that five tools among this suite are the focus of considerable policy debate, including flood maps, disaster assistance programs, flood insurance, disclosure and liability, and property buyouts. Against this backdrop, this study sought to understand public attitudes and expectations toward these five non-structural FRM instruments among Canadians living in high-risk areas.

### Flood Maps

Flood maps are spatial depictions of geographic areas that could be inundated by floods of various magnitudes, which are used to inform development decisions, plan for emergencies, and communicate flood risk to the public (Dransch et al. 2010; Hagemeier-Klose and Wagner 2009). Flood mapping in the Canadian federation is primarily the responsibility of the subnational provinces and territories, but the federal government has historically played a central role as well. For instance, the Flood Damage Reduction Program (FDRP) was an intergovernmental initiative that operated between 1975 and 1999, which sought to identify and map high-risk flood areas (de Loë 2000). This work resulted in 957 designated flood risk areas, most of which retain their designation today (Environment and Climate Change Canada 2013). After the discontinuation of the FDRP, flood mapping was largely decentralized to municipalities, but municipal-level flood maps are generally low quality and inaccessible to the public (Henstra et al. 2019). In recent years, there has been a renewed interest in flood maps, exemplified by the Flood Hazard Identification and Mapping Program, a federal initiative to fund the completion and updating of flood maps across the country (Natural Resources Canada 2022).

The social acceptability of flood maps is important because they are used to plan structural controls, guide development policies, and prioritize investments in risk reduction. Based on the considerations from existing scholarship cited above, the social acceptability of flood maps is likely to be mixed. On one hand, flood mapping distributes responsibility among multiple levels of government, while also placing an onus on households to use the maps for awareness and decision-making. On the other hand, flood maps are often contested by local governments and homeowners concerned that transparency about flood risk could reduce access to disaster assistance and reduce property values (Macdonald 2019; Raikes and McBean 2016).

### Disaster Financial Assistance

Disaster financial assistance aids affected communities and households in recovering from flood-related costs. Disaster assistance programs predominantly fund emergency response and recovery rather than prevention/mitigation and preparedness (Coppel and Chester 2014; Davies 2020). In Canada, both the federal and provincial governments operate disaster financial assistance programs. Provincial governments provide financial assistance to households to cover emergency expenses and costs to repair or replace essential property to a basic standard (Sandink et al. 2016). The Disaster Financial Assistance Arrangements (DFAA) is a federal cost-sharing program that reimburses provinces for disaster response and recovery costs that exceed a per-capita threshold (Public Safety Canada 2017). Disaster financial assistance programs have been under increased scrutiny in recent years, due to a federal policy change that raised the DFAA per-capita threshold (effectively reducing the federal contribution), the introduction of private flood insurance in 2015 (described below), and projections showing a dramatic increase in the Government of Canada’s future economic liability associated with flooding (Davies 2020).

As an instrument of FRM policy, disaster financial assistance is likely to have high social acceptability, because it shares with governments some of the economic liability that households would otherwise have to shoulder independently, it has no discernable impact on property values, and it places no controls on individual choices. However, citizens might not support financial relief if compensation is perceived as unfair or insufficient for recovery, or if the believe scarce public funds are being used to reward those who knowingly made risky choices (Husted and Nickerson 2021).

### Flood Insurance

Residential flood insurance covers property owners for flood losses in exchange for a premium. Flood insurance is regarded as an essential non-structural tool for FRM, because it offers a legitimate and efficient means to finance household recovery and it shares risk and responsibility beyond governments by engaging the private resources of insurers and property owners themselves (Sandink et al. 2016; World Meteorological Organization 2013). However, a viable flood insurance market requires both a supply of affordable coverage as well as sufficient demand from property owners (Netusil et al. 2021; Seifert et al. 2013).

Whereas property insurance in Canada has long covered losses associated with particular types of flooding, such as sewer back-up and water main breaks, it historically excluded overland flooding, which results from water seeping into buildings through windows, doors, and cracks (Meckbach 2017). Canadian insurers started to offer coverage for overland flooding in 2015, but its purchase is voluntary (Thistlethwaite 2017). Moreover, availability is variable across the country, with many property owners in high-risk flood areas unable to find affordable coverage (Golnaraghi et al. 2020). Additional barriers include the continued availability of disaster assistance (which limits incentives for property owners to purchase coverage), as well as inadequate flood risk awareness and uncertainty about the benefits of flood insurance, which manifest as a low willingness-to-pay for premiums (Thistlethwaite et al. 2020).

The social acceptability of flood insurance is important for its efficacy in markets like Canada’s, where insurance purchase is voluntary, rather than mandated by government. On one hand, private flood insurance should enjoy public support because it spreads responsibility across multiple stakeholders, including households, government and private firms, and it is non-coercive, in that individuals can choose whether or not to take it up. On the other hand, the social acceptability of private insurance as an FRM policy instrument could be undermined if some properties are considered too high-risk to be insurable or if coverage is available but only with unaffordable premiums.

### Flood Risk Disclosure and Liability

Flood risk disclosure refers to the mandatory release of information concerning a property’s flood history and/or its current risk when selling that property to a potential buyer. Liability refers to a legal responsibility imposed on a party to compensate another for damages linked to the former’s decisions or actions. In the context of flooding, for instance, legal liability could be attached to a party that caused or concealed flood risk and where it resulted in economic losses to another party.

Every provincial real estate association in Canada offers a form that asks sellers to disclose whether the property is subject to various conditions that could influence a potential buyer’s decision (Henstra and Thistlethwaite 2018). Completing the form is voluntary, however, and only Ontario’s Seller Property Information Statement includes a specific question about current flood exposure. In recent years, policymakers in Canada and other countries have shown a renewed interest in flood risk disclosure and liability as tools to raise awareness and share responsibility for FRM (e.g., Chopik 2019; Hersher and Sommer 2020), but these discussions have generally not included engagement with the public to gauge social acceptability.

Assessing the social acceptability of disclosure and liability is an important step toward determining their viability. On one hand, a disclosure and liability regime spreads responsibility for FRM beyond home buyers to include sellers, realtors, municipal governments, and possibly even lenders. On the other hand, adoption of this instrument set would place new controls on real estate transactions that the public might perceive as intrusive. Moreover, disclosure of flood risk information is often perceived to have negative impacts on property values (Inoue and Hatori 2021; Votsis and Perrels 2016) and liability is difficult to discern due to uncertainty around the causes and timing of flooding.

### Property Buyouts

Property buyouts seek to permanently reduce flood risk by relocating people out of high-risk areas through the public acquisition of land and property. Buyouts are typically implemented in response to recurrent disasters (particularly floods) and require a high upfront cost (Binder et al. 2015; Greer and Binder 2017). Although highly effective at reducing flood risk, buyouts often face opposition from homeowners unwilling to participate due to their attachment to place, economic prospects, and trust that the compensation offered is fair (Binder and Greer 2016).

Property buyout programs in Canada have historically been rare, limited in scope, and implemented reflexively in the aftermath of flooding, rather than grounded in thoughtful policy design (Saunders-Hastings et al. 2020). Political support for property buyout programs has been growing, however. In May 2019, for instance, several provinces approached the Government of Canada for more than $100 million to assist with acquisition of flood-prone properties (Press 2019). The same year, Canada’s national insurance industry association published a report that referred to buyouts as a “viable option” to manage flood costs of the highest risk residential properties (IBC 2019). In 2020, the Government of Canada committed funding to support managed retreat through property buyouts and launched a National Flood Insurance and Relocation Task Force to develop guidance on effective program implementation (Public Safety Canada 2020).

Gauging public attitudes toward property buyouts is instructive when considering the feasibility of their deployment or expansion as an instrument of FRM policy. Based on the considerations drawn from previous scholarship cited above, property buyouts should enjoy public support due to their spreading of economic liability to governments. However, existing literature suggests that this support would be contingent on the details of implementation, such as compensation rates and whether the buyouts were voluntary or mandatory.

## Methods

In June 2020, a bilingual, national survey was distributed to Canadian residents in high-risk flood areas. The survey consisted of 62 questions on a range of flood risk management themes, including flood maps, disaster financial assistance, flood insurance, disclosure and liability, and property buyouts. The survey was first piloted with 10 flood management experts and 25 property owners to identify weaknesses in the survey design. It was then distributed using a research firm that retains a panel of more than 300,000 Canadians, which are profiled based on more than 500 demographic, psychographic, behavioral, and attitudinal variables. With a target sample of 2300 participants and an expected incidence rate of 50%, the research firm used stratified random sampling to distribute the survey. The research firm was provided a list of Forward Sorting Area codes to assist in targeting residents in designated flood risk areas, as determined through the FDRP (Environment and Climate Change Canada 2013).

Overall, 2650 residents participated in the survey. Participants were distributed spatially across 9 of 10 Canadian provinces. Canadian territories were not included in this survey and the study yielded no significant participation from residents of Prince Edward Island. Participation in each of the nine provinces was proportional to that province’s population. This included 351 residents in British Columbia, 298 residents in Alberta, 99 residents in Saskatchewan, 116 residents in Manitoba, 986 residents in Ontario, 650 residents in Quebec, and collectively 150 residents of New Brunswick, Nova Scotia, and Newfoundland and Labrador (Table 1). Participation also varied by sociodemographic and socioeconomic characteristics such as age, income, house type, education, home value, years in home, homeowner/renter status, previous flood experience, and risk perception (Appendix A).

**Table 1:** Spatial distribution of surveyed participants by province

Province	Participation (*n* = 2650)
Ontario	37%
Quebec	25%
British Columbia	13%
Alberta	11%
Saskatchewan	4%
Manitoba	4%
Eastern Canada (New Brunswick, Nova Scotia, Newfoundland and Labrador)	6%

Responses to the survey were analyzed using descriptive statistics. Frequency distributions were calculated to determine participant convergence on each question. This process yielded results on public attitudes relating to each of the five policy instruments indicated above, including how participants view each instrument and the expectations associated with its use in FRM. Section Methods below reports the survey results.

## Results and Discussion

The national survey revealed several important insights on public attitudes toward policy instruments for FRM. This section focuses on those findings as they relate to (1) flood maps, (2) disaster assistance, (3) flood insurance, (4) disclosure and liability and (5) property buyouts. The section is followed by a broader discussion on the social acceptability of policy instruments for FRM.

### Flood Maps

Participants were asked to indicate their views on whether flood risk maps produced by governments should be publicly available and whether there should be provincial mandates to make maps public. Respondents indicated overwhelmingly that flood maps produced by governments should be publicly available to residents (90%) and that this should be mandated by provinces (89%). Moreover, 76% of participants indicated that the federal and provincial governments should provide special funding to smaller communities to assess flood risk and develop flood action plans.

Although flood mapping appeared to have strong social acceptability, most participants (81%) also indicated that they had not reviewed current flood maps for their community. This represents a significant challenge for flood mapping as a FRM policy instrument: though Canadians view flood maps as an important planning tool that should be accessible to the public, most people do not seek them out. As Handmer (2013) argued, flood maps can contribute to heightened awareness of flood risk but must be accompanied by public flood risk communication efforts.

The survey responses suggest that more user-friendly flood maps could garner more attention among citizens in order to raise awareness of flood risk. Experts argue effective public-facing flood maps should be searchable (e.g., by address or postal code), provide local context such as identifiable places or landmarks, designed to make it easy for the user to distinguish the extents of the flood hazard zone, transparent about limitations and uncertainty, and inclusive of all forms of flooding (e.g., Hagemeier-Klose and Wagner 2009; Merz et al. 2007; Van Alphen et al. 2009). These map features could increase public engagement with maps and hazard assessments, particularly as Canadian governments seek to motivate individuals to protect their property and purchase flood insurance (Thistlethwaite et al. 2018).

### Disaster Assistance

Survey participants were asked to indicate their views on the cost-sharing arrangements for flood disaster financial assistance, including who should be financially responsible for covering the costs of restoring a home to its pre-flood condition. Respondents indicated that multiple parties should share financial responsibility for recovery costs associated with flood damages and they distributed this perceived responsibility among FRM stakeholders. As Table 2 shows, most participants asserted that insurance companies and governments should bear most of the responsibility for paying for flood damages to residential properties, followed by homeowners. Furthermore, there was a dominant view among participants that non-governmental organizations should bear little responsibility for flood damage recovery costs.

**Table 2:** Responsibility for flood recovery costs

Stakeholder	Participant convergence (%)
Insurance company	76
Municipal government	36
Homeowners	34
Provincial government	34
Federal government	27
Non-governmental organizations	6

Percentage of participants reporting “very responsible” or “completely responsible”. Values rounded to nearest percentage

In addition, respondents varied in their perception of how much disaster assistance should be paid by governments to restore damaged homes to pre-flood conditions. As Table 3 shows, most participants indicated that governments should not be solely responsible for paying for damages to homes. Only 12% of participants indicated that governments should be responsible for paying for more than 80% of damages incurred from flooding. Together, the results from Tables 2 and 3 suggest that socially acceptable cost-sharing arrangements for disaster assistance would distribute costs between levels of government and among relevant stakeholders.

**Table 3:** Distribution of flood damage costs to homes that governments should be responsible for paying

Percentage of damages	Participant responses
0–20%	29%
21–40%	22%
41–60%	24%
61–80%	13%
81–100%	12%

Values were rounded to the nearest percentage

The results also highlight the path dependency associated with existing government-funded disaster assistance programs. Since Canadians expect governments to play a central role in disaster recovery, then it will be challenging to distribute the costs of recovery through insurance or alternative financial risk sharing schemes. The results also raise concerns about the feasibility of more proactive approaches that shift resources from recovery to pre-disaster mitigation, since citizens have an expectation that they will receive compensation for disaster damages through existing arrangements.

Through the Task Force on Flood Insurance and Relocation, the Government of Canada is leading an effort to expand private insurance to cover flood recovery costs, thereby reducing the government’s economic liability (Public Safety Canada 2020). This policy transition could be met with resistance if people in high-risk areas are unable to afford insurance and are less able to access public recovery funds. Moreover, any change in recovery cost-sharing arrangements that expects municipal governments to shoulder a greater share of the burden could falter due to the limited capacity of local governments to raise revenue.

### Residential Flood Insurance

Survey participants were asked if they had purchased flood insurance, whether homeowners in high-risk flood areas should be required to purchase flood insurance, what the costs of flood insurance should be, and whether premiums should be subsidized by governments or by other homeowners with lower flood risk. On the first question, only 23% of participants indicated that they had purchased insurance for overland flood damage, compared to 57% that had not. Moreover, 20% of the participants were unsure whether their existing homeowner insurance policy covered overland flood damage. Most participants (80%) indicated that homeowners should be required to purchase flood insurance if they live in a designated flood risk area, whereas only 5% asserted that buying flood insurance should be voluntary.

These findings offer guidance for the Government of Canada’s efforts to shape the flood insurance market. First, they reveal that voluntary purchase is unlikely to ensure sufficient market penetration, meaning the proportion of households within a geographic region that have taken up flood insurance. Indeed, if only a small group is aware whether they have flood insurance and many more are uncertain, any expansion of coverage might be exposed to adverse selection, whereby only the most at risk consider purchasing coverage. Private insurers are unlikely to participate in a market where adverse selection is prevalent since they will be exposed to recurring losses.

There is support for mandatory coverage among Canadians in high-risk areas, which would reduce adverse selection by broadening the pool of insured. But mandatory coverage is challenged by the fact that insurance market conduct in Canada is governed by provincial governments, which makes it difficult to harmonize policy across these ten different jurisdictions (CCIR 2008). Mandatory coverage is also likely to be resisted by insurers concerned that premiums could increase for lower-risk households, which would spur opposition from both property owners and governments.

Regarding the cost of flood insurance, 61% of respondents indicated that premiums should reflect the true risk to a property and should not be subsidized by governments or through the premiums paid by homeowners with lower flood risk. However, 52% of participants agreed that governments should subsidize premiums for lower income households, compared to 20% of participants who disagreed.

These results suggest two potential policy implications. First, Canada’s current effort to expand insurance coverage in high-risk areas will likely require that premiums from low-risk property owners are used to offset premiums in high-risk areas. The survey results suggest that this approach would need to be calibrated to ensure fairness by making those with the capacity to pay bear the full cost of insuring flood risk to their property. However, cross-subsidization of premiums to achieve broader affordability appears to be socially acceptable, so a second policy implication is that the approaches used in other countries to connect premiums with a household’s ability to pay (e.g., means-testing) might be feasible in Canada. In the United Kingdom, for example, flood insurance premiums are aligned with local council tax bands (Surminski and Eldridge 2014). In the United States, vouchers have been proposed as a means of subsidizing the high cost of flood insurance premiums for lower income households (Kousky and Kunreuther 2014).

### Disclosure and Liability

Participants overwhelmingly indicated that sellers (89%), realtors (90%), and municipalities (86%) should be required to inform potential buyers about a property’s flood risk if it is located in a designated flood risk area. This high level of support contrasts sharply with existing Canadian property disclosure regulations that rely on voluntary reporting of flood damage and risk. Moreover, 80% of participants indicated that a realtor should be liable for future flood damages if they sell a home without informing the buyer that the home has flooded previously. Similarly, 68% of respondents indicated that municipalities should be legally liable for flood damages if they approved a permit to build a home in a designated flood risk area.

These findings raise questions about the use of legal instruments to share, reduce, and manage flood risk. For example, whereas legal liability for flooding could make municipal governments more reluctant to approve development in high-risk areas, it could also prompt them to resist the release of new flood maps or to oppose the “high-risk” designation. This highlights the care needed to design an effective property disclosure regime, which would need to clarify the legal ambiguity over flood risk liability. Furthermore, assigning responsibility for flood risk disclosure to a third party could be necessary to increase its social acceptability because it removes the perception of bias surrounding sellers, realtors, and municipal governments (Henstra and Thistlethwaite 2018).

### Property Buyouts

Regarding property buyouts, about half of participants (49%) reported that residential properties at risk of recurrent flooding should qualify for public acquisition, compared to 20% of participants who disagreed. Participant responses indicated a series of conditions that should be incorporated into the design of property buyout programs, including voluntary participation (68%), fair compensation based on pre-flood market value or 75–100% of assessed value (55%), and a distribution of costs among municipal, provincial, and federal governments (59%). Participants also indicated that additional financial incentives should be built into program designs and buyout offers to subsidize relocation costs.

These findings are instructive for the design of future property buyout programs, suggesting that these initiatives should include flexible compensation options and cost-sharing arrangements to maximize social acceptability. Several Canadian local governments have implemented property buyout programs, including Gatineau in Quebec, Grand Forks in British Columbia, and High River in Alberta. All of these communities have faced resistance from homeowners opposed to moving from a location to which they are emotionally attached (Markusoff 2018). The survey findings mirror these experiences, in that mandatory participation is likely to be politically unpopular despite the risk reduction benefits of relocation.

## Social Acceptability of Policy Instruments for Flood Risk Management

The analysis of the survey results above offers broader insights into public attitudes toward FRM policy instruments and FRM more generally. First, communicating flood risk information is a crucial precursor to sharing, reducing, and managing flood risk. Participants in this study clearly indicated that they want access to flood risk information in the form of flood maps and mandatory property disclosure concerning flood risk. The fact that these respondents support these tools, despite living in designated flood risk areas, appears to challenge the notion in previous scholarship that homeowners will resist the release of this information out of fear of the potential consequences to their home value (e.g., Bakos et al. 2022; Inoue and Hatori 2021).

Second, survey participants favored a broader distribution of flood recovery costs, which suggests efforts to share flood risk among governments and a range of non-governmental stakeholders will enjoy high social acceptability. The DFAA, for example, shares disaster recovery costs between the federal and provincial governments, but this system could be reformed to assign some responsibility to other stakeholders, such as insurance companies and municipal governments. Any such change would require care, however, as the survey responses indicated strong continued support for a government-led approach. In alignment with previous scholarship, stakeholder engagement would be critical to maximize the social acceptability of any amendments to disaster financial assistance programs (Horney et al. 2016).

Third, FRM in high-risk flood areas should prioritize the protection of low-income households and support for municipalities with lower capacity to plan and implement FRM. Survey participants indicated that the federal and provincial governments should provide support to municipalities that lack the capacity to develop flood maps and corresponding emergency plans. Further, participants supported the idea that governments should provide subsidies for low-income households to obtain flood insurance. These findings also suggest a public expectation that governments will continue to play a significant role in FRM.

Fourth, there is clear support for the use of legal instruments to share, reduce, and manage flood risk. In particular, participants supported legal safeguards to make property buyers aware of flood risk, which is a common demand made of governments by victims and advocates in the aftermath of floods (Weisleder 2013). After major flooding in Alberta in 2005, for instance, a provincial flood mitigation committee recommended that “a notification system be established that will inform any potential buyer that the property is located within a designated flood risk area” (Groeneveld 2006, 3). However, the central policy design challenge is connecting disclosure with liability, which must be fairly and equitably distributed among key stakeholders. These stakeholders include municipal and provincial governments—which govern development in high-risk flood areas—but also developers, realtors, lenders, and property owners themselves.

Finally, although flood risk information is a cornerstone of effective FRM, information alone does not necessarily lead to risk reduction actions at the household level. The survey results suggest a tension between wanting better information about flood risk and a willingness to invest in property-level risk reduction (e.g., by reviewing flood maps or purchasing insurance). This suggests that the appropriate locus of FRM is the community scale rather than the individual property level. This approach has been effective in the United States, for example, where the Federal Emergency Management Agency administers a Community Rating System that offers financial rewards to households in communities that have invested in flood risk reduction (Sadiq et al. 2020).

Overall, the results indicate a significant shift in public attitudes around FRM, with greater public support for policy instruments that enable a more proactive approach. Canadian attitudes reflect an acknowledgement of FRM as an urgent policy problem requiring non-structural interventions. The survey responses also highlight public support for mechanisms that will enable property owners and residents to adopt risk reduction measures at the household level.

## Conclusion

Flooding is a significant global risk. A suite of strategies and a mix of supporting policy instruments is required to effectively share, reduce, and manage flood risk. In selecting policy instruments, governments must consider not only the projected effectiveness of these instruments in achieving the intended objectives, but also their social acceptability, which underpins their legitimacy and influences outcomes at the implementation stage.

Previous scholars have argued that high social acceptability fosters legitimacy and compliance with public policies (Drews and Bergh 2016), and this study complements those findings by providing insights into public attitudes toward policy instruments for FRM. Based on a national survey of residents in high-risk flood areas, the study evaluated the social acceptability of flood maps, disaster financial assistance, flood insurance, disclosure and liability, and property buyouts. The results show that all of these five instruments are socially acceptable, but they also highlight the ways in which these tools could be calibrated to maximize public support.

From a public policy perspective, the results indicate that the success of FRM requires a mix of policy instruments that informs residents about flood risk and reduces flood risk by sharing costs or relocating households. Flood maps offer potential to increase individual capacity to manage flood risk, while disaster assistance and insurance provide financial protection against flood damages. Legislation and regulations can prevent and mitigate flood risk, but they must be carefully designed to ensure fairness and clarify who should be liable for damages that result from individual choices.

While the study provides insights into public perceptions toward FRM policy instruments, further research is necessary. For instance, research on public perceptions of policy instruments for the management of other hazards could offer more insights into instrument design and implementation considerations. Whereas this study focused exclusively on the attitudes of residents in high-risk areas, a comparative study of people living in low-risk areas is also needed to further research insights into the social acceptability of FRM policy instruments. Moreover, a comparative analysis of other contexts (e.g., developing countries) or that specifically engages vulnerable groups (e.g., Indigenous communities) could advance this research and provide important insights to further evaluate the social acceptability of policy instruments in FRM. Finally, the social acceptability of the five FRM policy instruments as measured in this survey might reflect unique political values and beliefs held by Canadians that might not be congruent with those of citizens in other countries. The ways in which political and geographical contexts influence public attitudes toward FRM instruments should therefore be explored to further advance this field.

### Supplementary information

Supplementary Informartion
