# Supplementary material for: Public Attitudes Toward Policy Instruments for Flood Risk Management
Source: Environ Manage. 2023 Jul 1;72(5):1050–60. doi: 10.1007/s00267-023-01848-3 (PMC10509047; doi:10.1007/s00267-023-01848-3)
Supplement: Supplementary file 1 — Supplementary Informartion [file 267_2023_1848_MOESM1_ESM.docx]

# Appendix A

Table A.1: Participation by age group (n=2650)

| Age Range (years) | Number of Participants | Percentage of Sample Group |
| --- | --- | --- |
| 18-34 | 721 | 27% |
| 35-54 | 968 | 37% |
| 55+ | 961 | 36% |

Table A.2: Participation by income range (n=2650)

| Income Range ($ thousand) | Number of Participants | Percentage of Sample Group |
| --- | --- | --- |
| <30 | 178 | 7% |
| 30-49 | 279 | 11% |
| 50-99 | 880 | 33% |
| 100-149 | 538 | 20% |
| 150+ | 394 | 15% |
| Preferred not to answer | 381 | 14% |

Table A.3: Participation by house type (n=2650)

| House Type | Number of Participants | Percentage of Sample Group |
| --- | --- | --- |
| Detached home | 1796 | 68% |
| Semi-detached/townhouse | 457 | 17% |
| Apartment (low rise) | 218 | 8% |
| Condominium (low rise) | 110 | 4% |
| Other | 69 | 3% |

Table A.4: Participation by highest level of education completed (n=2650)

| Highest Level of Completion | Number of Participants | Percentage of Sample Group |
| --- | --- | --- |
| Primary School | 23 | 1% |
| Secondary/High School | 486 | 18% |
| College/Professional Trade Certificate | 789 | 30% |
| University – undergraduate degree | 802 | 30% |
| University – graduate/professional degree | 550 | 21% |

Table A.5: Participation by home value (n=2650)

| Home Value ($ thousands) | Number of Participants | Percentage of Sample Group |
| --- | --- | --- |
| <100 | 115 | 4% |
| 100-199 | 248 | 9% |
| 200-299 | 375 | 14% |
| 300-399 | 402 | 15% |
| 400-499 | 307 | 12% |
| 500-750 | 398 | 15% |
| >750 | 416 | 16% |
| Preferred not to answer | 389 | 15% |

Table A.6: Participation by years in current home (n=2650)

| Years | Number of Participants | Percentage of Sample Group |
| --- | --- | --- |
| <1 | 180 | 7% |
| 1-2 | 268 | 10% |
| 3-5 | 506 | 19% |
| 6-10 | 465 | 18% |
| 11-20 | 586 | 22% |
| >20 | 645 | 24% |

Table A.7: Participation by homeowner/renter status (n=2650)

| Status | Number of Participants | Percentage of Sample Group |
| --- | --- | --- |
| Homeowner | 2016 | 76% |
| Renter | 496 | 19% |
| Other | 138 | 5% |

Table A.8: Participants that have had experience with past flooding (n=2650)

| Experience | Number of Participants | Percentage of Sample Group |
| --- | --- | --- |
| Yes | 471 | 18% |
| No | 2179 | 82% |

Table A.9: Participants' concerns over future flooding (n=2650)

| Concern | Number of Participants | Percentage of Sample Group |
| --- | --- | --- |
| Not at all | 1244 | 47% |
| Slightly | 816 | 31% |
| Somewhat | 377 | 14% |
| Moderately | 172 | 6% |
| Extremely | 41 | 2% |
